# Territory-wide cohort study of Brugada syndrome in Hong Kong: predictors of long-term outcomes using random survival forests and non-negative matrix factorisation

**DOI:** 10.1136/openhrt-2020-001505

**Published:** 2021-02-05

**Authors:** Sharen Lee, Jiandong Zhou, Ka Hou Christien Li, Keith Sai Kit Leung, Ishan Lakhani, Tong Liu, Ian Chi Kei Wong, Ngai Shing Mok, Chloe Mak, Kamalan Jeevaratnam, Qingpeng Zhang, Gary Tse

**Affiliations:** 1 Cardiovascular Analytics Group, Laboratory of Cardiovascular Physiology, Hong Kong, China; 2 School of Data Science, City University of Hong Kong, Kowloon, Hong Kong; 3 Faculty of Medicine, Newcastle University, Newcastle upon Tyne, Tyne and Wear, UK; 4 Aston Medical School, Aston University, Birmingham, Birmingham, UK; 5 Tianjin Key Laboratory of Ionic-Molecular Function of Cardiovascular Disease, Department of Cardiology, Tianjin Institute of Cardiology, The Second Hospital of Tianjin Medical University, Tianjin, China; 6 Research department of Practice and Policy, University College London School of Pharmacy, London, UK; 7 Centre for Safe Medication Practice and Research, Department of Pharmacology and Pharmacy, The University of Hong Kong, Hong Kong, China; 8 Department of Medicine and Geriatrics, Princess Margaret Hospital, Hong Kong, Hong Kong; 9 Department of Pathology, Hong Kong Children's Hospital, Hong Kong, Hong Kong; 10 Faculty of Health and Medical Sciences, University of Surrey, Guildford, Surrey, UK

**Keywords:** ventricular fibrillation, ventricular tachycardia, arrhythmias, cardiac, electronic health records, biostatistics

## Abstract

**Objectives:**

Brugada syndrome (BrS) is an ion channelopathy that predisposes affected patients to spontaneous ventricular tachycardia/fibrillation (VT/VF) and sudden cardiac death. The aim of this study is to examine the predictive factors of spontaneous VT/VF.

**Methods:**

This was a territory-wide retrospective cohort study of patients diagnosed with BrS between 1997 and 2019. The primary outcome was spontaneous VT/VF. Cox regression was used to identify significant risk predictors. Non-linear interactions between variables (latent patterns) were extracted using non-negative matrix factorisation (NMF) and used as inputs into the random survival forest (RSF) model.

**Results:**

This study included 516 consecutive BrS patients (mean age of initial presentation=50±16 years, male=92%) with a median follow-up of 86 (IQR: 45–118) months. The cohort was divided into subgroups based on initial disease manifestation: asymptomatic (n=314), syncope (n=159) or VT/VF (n=41). Annualised event rates per person-year were 1.70%, 0.05% and 0.01% for the VT/VF, syncope and asymptomatic subgroups, respectively. Multivariate Cox regression analysis revealed initial presentation of VT/VF (HR=24.0, 95% CI=1.21 to 479, p=0.037) and SD of P-wave duration (HR=1.07, 95% CI=1.00 to 1.13, p=0.044) were significant predictors. The NMF-RSF showed the best predictive performance compared with RSF and Cox regression models (precision: 0.87 vs 0.83 vs. 0.76, recall: 0.89 vs. 0.85 vs 0.73, F1-score: 0.88 vs 0.84 vs 0.74).

**Conclusions:**

Clinical history, electrocardiographic markers and investigation results provide important information for risk stratification. Machine learning techniques using NMF and RSF significantly improves overall risk stratification performance.

**Key questions**

**What is already known about this subject?**

- Brugada syndrome (BrS) is an ion channelopathy that predisposes affected patients to spontaneous ventricular tachycardia/fibrillation (VT/VF) and sudden cardiac death. However, the epidemiology and risk factors in the Chinese patient population are not well-defined.

**What does this study add?**

- The main findings of this study are that (1) VT/VF incidence rate in Chinese subjects was similar between the present study and other cohort studies; (2) there was a significant difference in VT/VF incidence in patients with different initial disease manifestation; (3) ECG markers had significant variations between patients of different Brugada pattern and symptoms manifestation; (4) initial VT/VF presentation was a positive predictor for a shorter time before VT/VF occurrence during the follow-up in multivariate analysis, which is supported by the importance ranking of predictors generated from the random survival forest analysis.

**How might this impact on clinical practice?**

- Clinical and electrocardiographic risk factors are helpful for predicting ventricular arrhythmias in BrS. Machine learning techniques using random survival forest and non-negative matrix factorisation can further enhance risk prediction.

## Introduction

Brugada Syndrome (BrS) is a cardiac ion channelopathy that is characterised by abnormalities in action potential conduction and repolarisation. It predisposes affected individuals to the development of spontaneous ventricular tachycardia/fibrillation (VT/VF) and sudden cardiac death (SCD). While BrS has many forms of treatment, including the gold standard, implantable cardioverter-defibrillator (ICD) implantation, as well as conservative medical therapy or catheter ablation, neither strategy is considered perfect. In addition, the healthcare burden of BrS is further compounded by the lack of optimised risk stratification in the clinical setting, particularly among asymptomatic patients.

The prevalence of BrS worldwide displays significant regional heterogeneity. The influence of ethnicity and geographical location likely reflects variations in gene distribution, which collectively contribute to a comparatively higher incidence of BrS within Asian populations.^1^ However, despite its greater occurrence especially in Southeast Asia, the majority of the conducted cohort studies available in current literature are based in Western countries.^2–7^ The most notable multicentre study on BrS in Asia was based in Japan, with a primary focus on the relationship between BrS and mutations in SCN5A that encodes for the cardiac sodium channel pore-forming subunit,^8^ and long-term prognosis of 330 BrS patients.^9^ Earlier this year, a territory-wide screening for BrS was performed in Singaporean men as part of health screening before military service, which led to the identification of 287 individuals with confirmed Brugada patterns.^10^ Investigators from the Survey on Arrhythmic Events in Brugada Syndrome compared 364 White to 270 Asian BrS patients who had at least one ventricular arrhythmic event. They found that Asians presented almost exclusively as male adults and had a higher frequency of aborted cardiac arrests and spontaneous type 1 patterns.^11^

Given these findings of epidemiological difference in BrS, further understanding on the Asian BrS cohort is of critical importance. As such, we conducted this largest territory-wide BrS study in Asia, which aims to assess the clinical and electrocardiographic risk factors of SCD, and to evaluate the prognosis of Chinese BrS patients with different disease manifestations.

## Methods

### Study population

Due to its retrospective and observational nature, patient consent was waived by the committee. Patients were not involved in this study. The study conformed to the principles outlined in the Declaration of Helsinki. This study included consecutive patients diagnosed with BrS between 1997 to 2019 identified from searching electronic health records from the Hospital Authority of Hong Kong, as described previously.^12–15^ The diagnosis of BrS was confirmed by reviewing the patient case notes and documented ECGs by SL. and GT using the 2017 diagnostic criteria proposed by the Expert Consensus Statement.^16^ The joint guidelines from Heart Rhythm, European and Asian Society guidelines were adopted for the drug challenge test due to the use of older guidelines in past practice. The primary outcome of this study was spontaneous sustained VT/VF detected either during hospital admission or by ICD data. The predictive value of baseline ECG parameters was explored for all patients, while the SD and the average of the ECG parameters over time were explored for patients with more than one ECG. SD of ECG parameters was included as predictors to examine the prognostic value of ECG variability. Further details and methods of statistical analysis, including Cox and Random survival forest (RSF) analysis are shown in online supplemental appendix.

10.1136/openhrt-2020-001505.supp1 Supplementary data

Non-negative matrix factorisation (NMF) represents a group of algorithms in the multivariate analysis and linear algebra with the property that all three matrices have no negative elements.^17^ First, we constructed matrix V representing the interrelations among the risk predicctors (eg, age of initial presentation, female gender, initial syncope, etc). Second, NMF decomposes matrix V into a core matrix W multiplied by a matrix H with different component cases (ie, number of latent variables generated). The generated latent variables were then combined with the risk predictors as the input for the RSF model. Prediction performance was evaluated by metrics of precision, recall and F1-score. The NMF module in the scikit-learn package (V.0.23.2) in Python was used. The RSF model can be automatically computed using the R-package randomForestSRC (V.2.9.3).

## Results

### Baseline characteristics

The study cohort consists of 516 consecutive patients (mean age of initial presentation=50±16 years, male=92%) with a mean follow-up period of 87±53 months (IQR=(45–118) months). A total of 2715 ECGs were analysed. 75% patients presented with a type 1 Brugada pattern (BrP), and evolution in BrP occurred in 34% patients. Family history of BrS and SCD is present in 3% and 8% of the cohort, respectively, with no significant intergroup differences. 16% patients have concomitant arrhythmia of other types. Investigations, including sodium channel blocker challenge (n=198, positive=88%), EPS studies (n=112, positive=68%), 24-hour Holter study (n=140, positive=44%), treadmill exercise tolerance test (n=63) and echocardiogram (n=57). ICD was implanted in 136 patients, with appropriate shocks received by 37 patients and 30 patients experienced inappropriate shocks. Within the secondary prevention group, 16% patients did not receive an ICD due to personal choice for reasons such as financial concerns. Genetic tests were performed for 10% of the cohort, with only 32% tested positive. An electroencephalogram (EEG) was performed under clinical suspicion of seizure in 11% of the cohort, with abnormal waveforms found in 28.1% of those investigated. A total of 448 patients have ECGs with automatically measured ECG parameters from at least one ECG, and 267 patients with more than one ECG with measured parameters taken on separate days.

### Analysis based on initial symptoms

**Figure 1:**
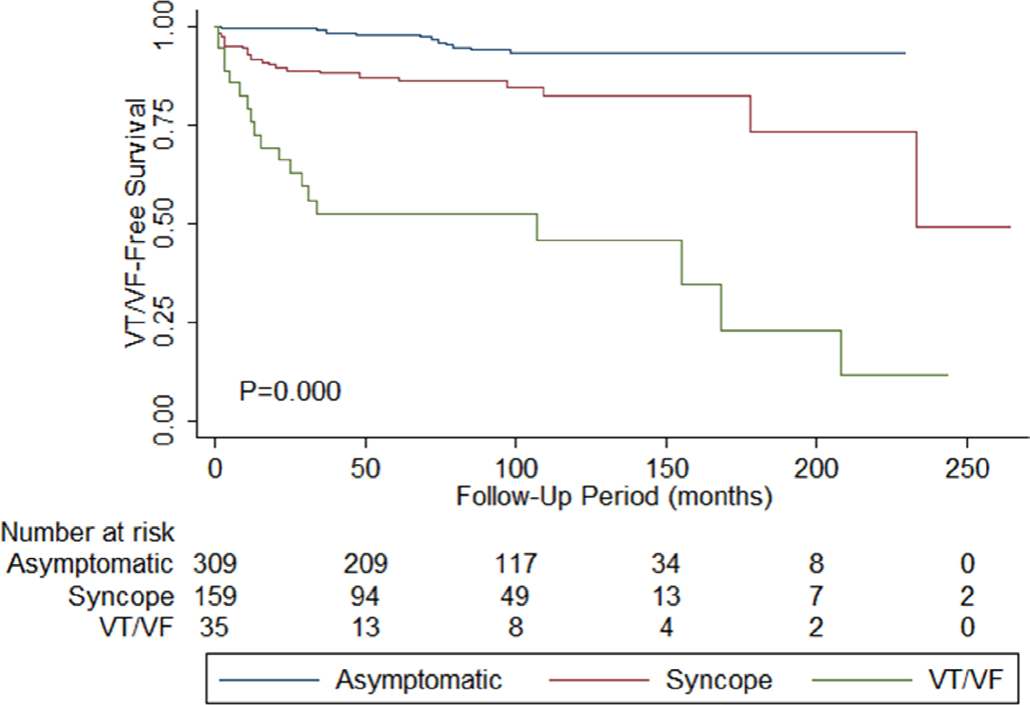
Kaplan-Meier curves demonstrating freedom from spontaneous ventricular tachycardia/ventricular fibrillation (VT/VF) during the follow-up for the initially asymptomatic (blue), syncope (red) and VT/VF (green) groups. Total size of cohort: n=516. A p<0.001 by the log-rank test.

Patients were compared based on disease presentation at initial BrP presentation: (1) asymptomatic (n=314, initial BrP presentation age=51±16, follow-up duration=86±53 months); (2) syncope (n=161, initial BrP presentation age=49±17, follow-up duration=87.2±52 months); (3) VT/VF (n=41, initial BrP presentation age=46±18, follow-up duration=90±64 months). The baseline characteristics are presented in table 1. The mean VT/VF event rate per person-year differed significantly (p<0.0001), in descending order of VT/VF (1.70%), syncope (0.05%) and asymptomatic (0.01%). There is a significant intergroup difference in the time till VT/VF occur during follow-up (figure 1; p value: asymptomatic vs syncope <0.0001, asymptomatic vs VT/VF<0.0001, syncope vs VT/VF<0.0001), the shortest time being the VT/VF group, followed by syncope and the asymptomatic group.

**Table 1:** Baseline characteristics of patient subgroups based on initial symptoms presented

Feature	Initial asymptomatic (n=315)	Initial syncope (n=159)	Initial VT/VF (n=42)	P value
Female	22 (6.98)	15 (9.43)	2 (4.76)	0.567
Age of initial presentation	51.0±15.8	49.0±16.5	45.8±17.9	0.113
Follow-up period (months)	85.5±52.8	88.0±52.0	89.4±63.1	0.873
Initial type 1 BrP	202 (64.1)	95 (59.7)	22 (52.4)	0.280
Type 1 BrP	247 (78.4)	114 (71.7)	27 (64.3)	0.065
Evolution	117 (37.1)	47 (29.2)	13 (31.0)	0.236
Fever induced	59 (18.7)	17 (10.7)	3 (7.14)	**0.024**
Family history of BrS	13 (4.13)	2 (1.26)	1 (2.38)	0.211
Family History of VF/SCD	26 (8.25)	12 (7.55)	3 (7.14)	0.967
Syncope	35 (11.1)	159 (100)	28 (66.7)	**<0.0001**
Number of syncope	1.37±21.6	2.01±2.56	0.905±0.821	**<0.0001**
VT/VF	13 (4.13)	25 (15.7)	42 (100)	**<0.0001**
Number of VT/VF episodes	0.302±3.14	1.39±7.86	8.10±14.7	**<0.0001**
Mean VT/VF event rate ratio per person-year, %	0.010	0.048	1.70	**<0.0001**
High VT/VF burden	3 (0.952)	12 (7.55)	22 (52.4)	**<0.0001**
Drug challenge performed	121 (38.4)	82 (51.6)	22 (52.4)	**0.012**
Drug positive*	109 (90.1)	72 (87.8)	17 (77.3)	0.245
EPS performed	50 (15.9)	53 (33.3)	9 (21.4)	**<0.0001**
EPS positive*	28 (56.0)	38 (73.6)	9 (100)	**0.014**
ICD	35 (11.4)	66 (41.5)	34 (81.0)	**<0.0001**
Holter performed	89 (28.3)	44 (27.7)	7 (16.7)	0.285
Abnormal Holter*	36 (40.4)	21 (47.7)	4 (57.1)	0.531
Other arrhythmias†	39 (12.7)	28 (17.6)	12 (28.6)	**0.022**
Genetic test performed	27 (8.60)	15 (9.43)	9 (21.4)	0.052
Genetic test positive*	9 (33.3)	4 (16.0)	3 (33.3)	0.922
Treadmill performed	41 (13.0)	18 (11.3)	4 (9.52)	0.794
Echocardiogram performed	138 (43.8)	73 (45.9)	26 (66.7)	**0.020**
EEG performed	13 (4.13)	33 (20.1)	12 (28.6)	**<0.0001**
EEG positive	3 (23.1)	5 (15.2)	8 (66.7)	**<0.0001**

P-values less than 0.05 are shown in bold text.

*Indicates the patient percentage within the subgroup population where the investigation is performed

†Other arrhythmias include sick sinus syndrome, bradycardia, atrioventricular block, atrial tachyarrhythmias and supraventricular tachyarrhythmias.

BrP, Brugada pattern; BrS, Brugada syndrome; EEG, electroencephalogram; EPS, electrophysiological study; ICD, implantable cardioverter-defibrillator; SCD, sudden cardiac death; VF, ventricular fibrillation; VT, ventricular tachycardia.

Both average (p value: asymptomatic vs syncope=0.278, asymptomatic vs VT/VF=0.015, syncope vs VT/VF=0.042) and baseline QTc interval (p value: asymptomatic vs syncope=0.280, asymptomatic vs VT/VF=0.008, syncope vs VT/VF=0.033) are significantly longer in patients who presented with VT/VF initially. The SD of QRS duration differed significantly by the descending order of VT/VF (11.5±12.4 ms), asymptomatic (8.13±9.17 ms) and syncope (6.35±5.12 ms) (p=0.004). The SD in the T-wave axis is significantly higher for the VT/VF group (p value: asymptomatic vs syncope=0.346, asymptomatic vs VT/VF=0.042, syncope vs VT/VF=0.013). On the contrary, the T-wave axis at baseline is the lowest for the VT/VF group (p value: asymptomatic vs syncope=0.508, asymptomatic vs VT/VF=0.017, syncope vs VT/VF=0.044). Follow-up and predictors of spontaneous VT/VF outcomes postdiagnosisIn total, 71 patients suffered from spontaneous VT/VF in our cohort. Of these, 62 patients showed spontaneous VT/VF during the follow-up. The overall mean VT/VF incidence rate ratio per person-year is 0.004%. Thirteen patients were excluded from the analysis since they were initially cardiac event-free and prescribed quinidine. Univariate Cox regression analysis for predictors of shorter time to first post-diagnosis VT/VF episode are presented in table 2. The following significant parameters were identified: (1) symptomatic at diagnosis (HR=5.18, 95% CI=2.69 to 9.96, p≤0.0001); (2) VT/VF at diagnosis (HR=11.3, 95% CI=6.32 to 20.3, p<0.0001); (3) syncope at diagnosis (HR=2.24, 95% CI=1.11 to 4.53, p=0.025); (4) concomitant presence of other arrhythmia (HR=3.02, 95% CI=1.67 to 5.45, p=<0.0001); (5) average QRS duration (HR=1.02, 95% CI=1.00 to 1.03, p=0.027); (6) average QTc interval (HR=1.01, 95% CI=1.00 to 1.02, p=0.013); (7) P wave duration SD (HR=1.04, 95% CI= 1.00 to 1.09, p=0.033); 8) QRS axis SD (HR=1.01, 95% CI= 1.00 to 1.02, p=0.048)and 9) baseline QTc interval (HR=1.01, 95% CI=1.00 to 1.02, p=0.022). The optimum VT/VF-protective cut-offs for QRS, QTc and QT were 109.8 ms, 419.6 ms and 364.9, respectively.

**Table 2:** Univariate Cox regression for predictors of shorter time to VT/VF postdiagnosis

Feature	HR	95% CI	P value
Female	0.219	(0.030 to 1.59)	0.133
Age of initial presentation	1.00	(0.983 to 1.02)	0.874
Initial syncope	2.24	(1.11 to 4.53)	**0.025**
Initial VT/VF	11.3	(6.32 to 20.3)	**<0.0001**
Initial type 1 BrP	0.981	(0.551 to 1.75)	0.949
Evolution	0.562	(0.296 to 1.07)	*0.078*
Fever	0.435	(0.135 to 1.40)	0.163
Family History of BrS	0.536	(0.074 to 3.90)	0.538
Family History of VF/SCD	0.845	(0.262 to 2.72)	0.778
Other arrhythmia	3.02	(1.67 to 5.45)	**<0.0001**
EPS positive	3.29	(0.954 to 11.3)	*0.059*
Abnormal Holter	2.85	(0.835 to 9.74)	*0.095*
Genetic positive	0.504	(0.135 to 1.88)	0.308
Average			
Heart rate	1.01	(0.992 to 1.02)	0.324
PWD	1.00	(0.981 to 1.03)	0.732
PR interval	0.999	(0.987 to 1.01)	0.858
QRS duration	1.02	(1.00 to 1.03)	**0.027**
QT interval	1.00	(0.995 to 1.01)	0.332
QTc Interval	1.01	(1.00 to 1.02)	**0.013**
P axis	1.01	(0.982 to 1.03)	0.616
QRS Axis	0.997	(0.990 to 1.00)	0.307
T Axis	1.00	(0.988 to 1.01)	0.877
V5 R wave amplitude	0.895	(0.459 to 1.75)	0.745
V1 S wave amplitude	0.519	(0.157 to 1.71)	0.282
SD			
Heart rate	1.01	(0.966 to 1.05)	0.780
PWD	1.04	(1.00 to 1.09)	**0.033**
PR interval	1.01	(0.984 to 1.03)	0.510
QRS duration	1.02	(0.988 to 1.05)	0.247
P axis	1.01	(0.982 to 1.03)	0.616
QRS axis	1.01	(1.00 to 1.02)	**0.048**
T axis	1.00	(0.982 to 1.02)	0.730
V5 R wave amplitude	1.37	(0.192 to 9.79)	0.752
V1 S wave amplitude	2.29	(0.053 to 99.7)	0.666
Baseline			
Heart rate	1.01	(0.990 to 1.02)	0.489
PWD	1.01	(0.982 to 1.03)	0.668
PR interval	1.00	(0.989 to 1.01)	0.879
QRS duration	1.00	(0.992 to 1.01)	0.580
QT interval	1.00	(0.997 to 1.01)	0.255
QTc interval	1.01	(1.00 to 1.02)	**0.022**
P axis	0.998	(0.985 to 1.01)	0.788
QRS axis	0.999	(0.993 to 1.00)	0.713
T axis	1.00	(0.989 to 1.01)	0.989
V5 R wave amplitude	0.632	(0.296 to 1.35)	0.236
V1 S wave amplitude	0.419	(0.105 to 1.67)	0.219

P-values less than 0.05 are shown in bold text.

BrP, Brugada pattern; BrS, Brugada syndrome; EPS, electrophysiological study; PWD, P-wave duration; QTc, corrected QT interval; SCD, sudden cardiac death; VF, ventricular fibrillation; VT, ventricular tachycardia.

Multivariate analysis showed that initial VT/VF (HR=24.0, 95% CI= 1.21 to 479, p=0.037) and SD of P-wave duration (HR=1.07, 95% CI=1.00 to 1.13, p=0.044) were predictive of spontaneous VT/VF (table 3). Initial symptomatic presentation was excluded as a predictor since it includes the subset of patients presented with VT/VF initially. Mortality statistics are shown in online supplemental appendix.

**Table 3:** Multivariate Cox regression analysis for clinical and ECG predictors of shorter time to VT/VF postdiagnosis

Feature	HR	95% CI	P value
Initially VT/VF	24.0	(1.21 to 479)	**0.037**
Initial syncope	7.19	(0.622 to 83.1)	0.114
Other arrhythmia	1.04	(0.141 to 7.70)	0.969
QRS duration average	1.06	(0.975 to 1.16)	0.171
QTc interval average	0.997	(0.957 to 1.04)	0.874
P wave duration SD	1.07	(1.00 to 1.13)	**0.044**
QRS axis SD	1.01	(0.990 to 1.03)	0.364

P-values less than 0.05 are shown in bold text.

QTc, corrected QT interval; VF, ventricular fibrillation; VT, ventricular tachycardia.

Focusing on primary prevention, excluding patients with prior VT/VF events, a total of 474 patients were analysed. Of these, 36 suffered from spontaneous VT/VF on follow-up. For this cohort, univariate Cox regression revealed initial presentation with syncope (HR: 3.94, 95% CI 1.96 to 7.92; p<0.0001), presence of other arrhythmias (HR: 2.93, 95% CI 1.44 to 5.95, p=0.003), average QRS (HR: 1.03, 95% CI 1.01 to 1.05; p=0.002) and QTc (HR: 1.01, 95% CI1.00 to 1.03, p=0.047) were significantly associated with incident VT/VF development. Interestingly, an evolution of BrP was a protective factor (HR: 0.44, 95% CI0.20 to 0.96; p=0.039).

### RSF and NMF analysis

**Figure 2:**
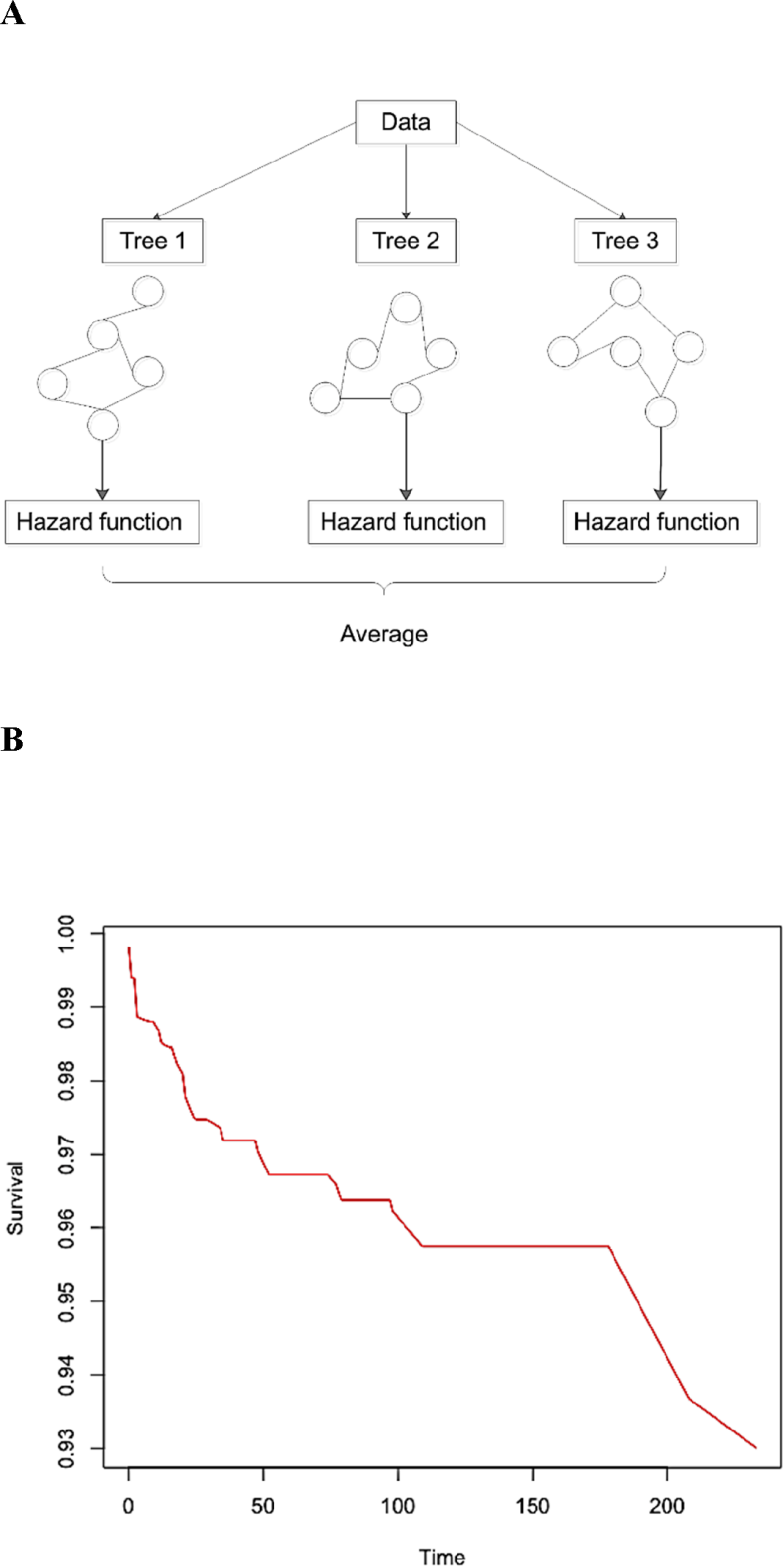
Principles of the random survival forest model (A). Features and samples are randomly selected for each single tree, and log-rank splitting is used to grow the trees. At the end of each branch, a cumulative hazard function is calculated for the selected individual trees. Finally, the ensembled estimated cumulative hazard function is computed by averaging the results of all the trees. survival curve from the random survival forest model (B).

The principles of RSF analysis are summarised in figure 2A. The importance attached to each variable assessed is shown in table 4 and the survival curve generated by the RSF model is shown in figure 2B. The generated importance ranking of risk predictors (continuous or categorical) can be used by clinicians to infer the mortality probability through checking those that were highly ranked (ie, demonstrate great importance in the prediction). We found that the RSF model significantly outperformed the Cox regression model (table 5). The data input into the RSF model is shown in online supplemental table 1. Sensitivity analysis was performed by excluding the genetic test (online supplemental table 2), electrophysiological study (online supplemental table 3) or both genetic test and electrophysiological study (online supplemental table 4).

**Table 4:** Importance of different variables used in random survival forest analysis

Variable	Importance	Rank
Prior VT/VF	0.3120	1
Syncope or spontaneous VT/VF	0.0289	2
Age	0.0055	3
QTc interval	0.0039	4
QRS axis	0.0030	5
Syncope	0.0024	6
P wave axis	0.0015	7
QT interval	0.0014	8
T wave axis	0.0012	9
QRS interval	0.0008	10
SV1 amplitude	0.0004	11
PR interval	0.0002	12
P wave duration	0.0001	13
Sex	0.0001	14
Positive genetic test	0.0001	15
Fever induced type 1	0.0000	16
Ventricular rate	0.0000	17
Initial type 1 Brugada pattern	0.0000	18
Brugada pattern evolution	0.0000	19
Family history of Brugada syndrome	−0.0001	20
Positive electrophysiological study	−9.02E-05	21
RV5 amplitude	−1.98E-04	22
Family history of sudden cardiac death	−2.09E-04	23
Positive Holter findings	−2.12E-04	24
Presence of other arrhythmias	−2.78E-04	25
Fever	−3.18E-04	26

QTc, corrected QT interval; RV5, R-wave amplitude in V5; SV1, S-wave amplitude in lead V1; VF, ventricular fibrillation; VT, ventricular tachycardia.

**Table 5:** Out-of-sample (fivefold cross-validation) performance comparisons among Cox model, RSF model and RSF-NMF model with all variables

	Precision	Recall	F1-score
Cox model	0.7565	0.7280	0.7420
RSF model	0.8338	0.8531	0.8433
RSF-NMF model	**0.8712**	**0.8881**	**0.8796**

P-values less than 0.05 are shown in bold text.

NMF, non-negative matrix factorisation; RSF, random survival forest.

Further, a total of five latent patterns (referred to as latent 1–5) were extracted by NMF on the collected risk predictors according to the sensitivity analysis results of latent variable extraction by the RSF-NMF model (table 6). Incorporation of the extracted five latent variables into the RSF model achieved the best prediction performance overall in a out-of-sample five-fold cross-validation approach (table 5) (precision: 0.87, recall: 0.89, F1-score: 0.88).

**Table 6:** Sensitivity analysis of latent variables by the RSF-NMF model

No of latent variables	3	4	5	6	7	8
Precision	0.8145	0.8529	**0.8949**	0.8705	0.8804	0.8203
Recall	0.7934	0.8499	**0.8756**	0.8671	0.8704	0.8025
F1-score	0.8038	0.8514	**0.8851**	0.8688	0.8754	0.8113

P-values less than 0.05 are shown in bold text.

NMF, non-negative matrix factorisation; RSF, random survival forest.

## Discussion

This is one of the largest territory-wide cohort studies on BrS and the largest study in Asia published to date, with an extensive median follow-up duration of 7 years. The main findings of this study are that (1) VT/VF incidence rate was similar between the present study and other cohort studies; (2) there was a significant difference in VT/VF incidence in patients with different initial disease manifestation; (3) ECG markers had significant variations between patients of different BrP and symptoms manifestation and (4) initial VT/VF presentation was a positive predictor for a shorter time before VT/VF occurrence during follow-up in multivariate analysis, which is supported by the importance ranking of predictors generated from the RSF analysis.

### Epidemiological and geographical differences in prevalence and event rates

BrS has a high prevalence in Asia with a five-fold difference compared with western populations.^1^ A recently published systematic review and meta-analysis found that the prevalence is the highest in Southeast Asia, followed by North Africa, Middle East, East Asia, South Asia, North America, Europe and Hawaii.^1^ However, epidemiological and particularly outcomes-based data from Asia on BrS are lacking.^18^ A recent study found that there was a surprisingly low prevalence of spontaneous type 1 Brugada pattern in young Singaporean men, but this may be due to age-related penetrance.^10^ Not all patients with BrS will eventually suffer from spontaneous VT/VF during their lifetime. In our study, 12% of the patients exhibited spontaneous VT/VF after diagnosis and 16% of the whole cohort showed VT/VF overall. The overall mean VT/VF incidence rate ratio per person-year is 0.004% using individual patient-level data. Using the number of individuals and mean follow-up duration across the population, this yielded an incidence rate ratio of 0.004% in our study, which similar to figures reported by the France, Italy, Netherlands, Germany (FINGER) registry after conversion to incidence rate ratio (0.001%).^2^ For the Singaporean cohort, none of the subjects exhibited spontaneous VT/VF after 2 years of follow-up.^10^ In the multicentre Japanese study, 19 of the 330 BrS patients had arrhythmic events over 48.7 months of follow-up.^9^

### Predictors of spontaneous VT/VF: Holter, genetic screening, ECG variables and EPS

The event rates of spontaneous VT/VF differ depending on initial symptoms, with prior VT/VF/SCD, syncope and asymptomatic subgroups. Results from the multi-centre FINGER registry reported annual event rates of 7.7%, 1.9% and 0.5%, respectively.^19^ For the multicentre Japanese study, these rates were 10.2%–10.6%, 0.6%–1.2% and 0–0.5.^9^ In a cohort of 90 Thailand BrS patients, the values were 6.7%, 6.0% and 0%.^20^ In our study, the rates were comparable with values of 7.83%, 2.22% and 0.62%. We found that patients who were initially symptomatic (syncope/VT/VF), syncope, those with prior VT/VF and those suffering from other arrhythmias (eg, atrial tachyarrhythmias) were at significantly higher risk of future events. Those 314 BrS patients who were initially asymptomatic, 14 nevertheless went on to develop spontaneous VT/VF. Interestingly, four of these 14 patients developed syncope during their disease life course, indicating the importance of symptom reassessment for ongoing risk stratification. Of these four patients, two had a family history of BrS, with one showing a type 1 pattern and the other a type 2 pattern. In the two remaining patients, one patient only suffered from VF when suffering from fever with chest sepsis, whereas the other had inducible polymorphic VT during EPS and a shock for VT/VF after ICD implantation. Prior studies have also found a low but nevertheless elevated risk of ventricular arrhythmogenesis in the asymptomatic group. Recently, Letsas *et al* reported that out of the 75 asymptomatic BrS patients, one suffered from VT/VF during the follow-up, which corresponded to an annual incidence of 0.3% per year.^19^ Annual incidences of this asymptomatic group were 0.5% from the FINGER registry (n=654),^2^ 0.8% per year by Delise *et al* (n=320;),^4^ 0.6% by Sieira *et al* (n=269)^5^ and 0.04% in Kamakura *et al* (n=154).^9^

Moreover, the initial presentation of type 1 BrP and type 1 BrP observed at any point during follow-up were not identified as significant predictors of spontaneous VT/VF. This is most likely due to the unusually high incidence of type 1 BrP in the entire cohort, which may have blunted the intergroup difference in spontaneous VT/VF occurrence. The manifestation of syncope lost its predictive value under multivariate Cox regression analysis is likely due to the presence of possible non-cardiogenic syncope and patient under-reporting. Furthermore, there was not a significant difference in the total VT/VF incidence rate between drug-induced (n=83) and spontaneous type 1 BrP patients (n=280), with the exclusion of those who had drug-induced type 1 BrP and evolved into type 1 BrP spontaneously (n=25) (drug-induced type 1=0.071% vs spontaneous type 1=0.073%, p=0.876).

Beyond the type of BrP, sinus node status, the co-occurrence of other arrhythmias, depolarisation and repolarisation markers have also demonstrated predictive value for risk stratification.^21–24^ For example, the multicentre international study by Delinière *et al* found that maximum corrected T_peak_-T_end_ intervals≥100 ms in precordial leads, type 1 Brugada pattern in a peripheral lead, early repolarisation in inferolateral leads, and QRS duration ≥120 ms in lead V2 were important risk variables. From the study by Sieira *et al* (n=269), QRS duration and sinus node dysfunction were significant predictors, with atrial fibrillation showing a borderline significance.^5^ QRS in lead V2 ≥113 ms and fragmented QRS complexes were identified as significant predictors by Letsas *et al*,^19^ whereas S-wave (≥0.1 mV and/or≥40 ms) in lead I was proposed by Calò *et al*.^25^ These studies illustrate the importance of depolarisation abnormalities in the form of slowed conduction and increased heterogeneity in conduction in promoting arrhythmogenesis. The present study also found that P wave duration SD and the presence of other arrhythmias such as AF, mean QRS duration and QTc intervals were predictors of spontaneous VT/VF. Our findings, therefore implicate an additional role for atrial arrhythmias and abnormalities in ventricular repolarisation as important determinants of ventricular arrhythmogenesis in BrS and ECG biomarkers reflecting such processes provide incremental value for risk stratification.^26^

However, in our study, given that QRS duration was a predictive factor in univariate analysis, it may have also contributed to the significance of QTc duration. It was, therefore. not possible to separate the relative contributions between depolarisation and repolarisation abnormalities to the arrhythmic substrate in this study. Additionally, the significantly smaller degree in T wave axis in the VT/VF group may be a reflection of smaller QRS amplitude and horizontal ST segments in right-sided leads, which have been reported as risk factors for VT/VF.^27 28^

It is known that the BrP can fluctuate over time even in an absence of precipitating factors such as fever.^29 30^ Therefore, the assessment of ECG variables and EPS status in the temporal domain may provide additional value for risk stratification beyond single ECGs. In our study, we found that 318 patients initially had a spontaneous type 1 pattern. However, an additional 70 patients were identified as having a type 1 pattern on subsequent ECG analyses. These findings are in keeping with the data from the Brugada group, who found that in BrS patients with spontaneous coved-type ECG, only every third ECG was diagnostic and every third ECG was normal.^31^ Signal-averaged ECGs can reveal late potentials that are associated with conduction abnormalities and ventricular arrhythmogenesis.^32^ Moreover, arrhythmic findings in 24-hour Holter monitoring were significantly predictive. EPS data obtained over a period of time may also provide additional prognostic value. Thus, Gray *et al* assessed the spatial burden defined as the number of precordial leads demonstrating diagnostic ST-segment elevation, and temporal burden defined as the number of 5 min time points demonstrating diagnostic ST-segment elevation.^33^ These authors found that a high temporal burden was associated with adverse cardiac events. In our study, a high SD of QTc interval measured over serial ECGs was a significant predictor of incident spontaneous VT/VF, indicating that temporal variability in repolarisation is another proarrhythmic substrate. Together our analysis demonstrates the value of serial ECG assessment in risk stratification.

EPS is the hallmark test for risk stratification in BrS and other proarrhythmic conditions. In our study, the positive EPS test was a significant predictor of subsequent spontaneous VT/VF episodes. However, 5 out of 36 patients with a negative EPS test nevertheless went on to develop spontaneous VT/VF. Only 21 out of 76 patients (28%) with a positive EPS had spontaneous VT/VF on follow-up. In other words, 72% of these patients are actually ‘low-risk’ patients. These findings suggest that EPS outcome alone is not a good deciding factor for determining whether patients are at a high or low risk of arrhythmogenesis. Out of the 22 patients who were both asymptomatic and EPS negative, none had developed spontaneous VT/VF. Therefore, this category of BrS patients appears to be truly at low risk of VT/VF and SCD.

### Application of machine learning to improve risk prediction

RSF builds hundreds of trees and generates outcome prediction by voting method for analysing right censored survival data.^34^ The advantage is that unlike the Cox proportional hazard model, it does not make assumptions about the individual hazard function^35^ and ranks the significance of predictors for spontaneous VT/VF. The advantage of RSF is that the boosting tree structure can capture the nonlinear effects and complex interactions among the variables, which can reduce prediction variance and bias, and improve learning performance.^35^ RSF was shown to improve predictive performance for sudden cardiac arrest events in the left ventricular structura predictors of SCD Registry^36^ and ventricular tachyarrhythmias in congenital long QT syndrome.^37^

NMF represents a group of algorithms used for dimensional reduction and feature extraction on non-negative data.^17^ This permitted hidden features between risk variables to be identified. This non-negativity makes the resulting matrices easier to inspect and makes the interpretation easier for real-world applications, such as identification of hidden stages in embryonic stem cell differentiation,^38^ DNA methylation profiling of human cardiac tissue^39^ and unsupervised cf-mRNA transcriptome decomposition.^40^ NMF was recently used by our teams for mortality risk prediction in acquired long QT syndrome patients^41^ and arrhythmic risk stratification in BrS patients.^42^ In this study, these latent factors were then used as inputs an RSF model. We showed that the combined NMF-RSF model provided the best time-to-event outcome predictions, when compared with RSF and Cox regression models.

### Limitations

Several limitations should be noted for the present study. First, this is a retrospective study and may be subjected to certain types of bias. However, there were at least 6 monthly to annual consultations for most patients, and therefore, follow-up information was excellent. Moreover, if patients are admitted to hospitals other than their usual hospital, their case records can be tracked by linked electronic health records. Second, the predictive value of investigations was limited by the relatively small sample size of patients with the investigations performed, despite being the largest cohort in Asia, and may be affected by the indications. Thirdly, syncope could be of non-cardiogenic origin and potentially unrelated to BrS. Fourth, the evolution in guidelines for EPS and genetics testing over the course of the follow-up period result in inevitable inconsistency in guidelines adopted by clinicians, given that the recommended protocol for EPS was modified several times over the past 10–15 years. The precise protocol used may differ between institutions, which contributed to the discrepancy in what was considered a positive outcome. Fifthly, the automated ECG measurements were averaged from the 12 leads, hence cannot reflect the difference in variation in each lead, in particular the right precordial leads. Sixth, regarding the SD of ECG variables, this could not be calculated for every patient as some patients only had one ECG available for analysis. This might have introduced inadvertent bias to the analyses. Therefore, the predictive value of the SD of P-wave duration remains to be confirmed in future studies. Finally, given the reliance on case records or the absence of some ECGs for the ventricular arrhythmic episodes, it was not possible to further distinguish between monomorphic and polymorphic VT.

## Conclusion

Clinical history, ECG markers and investigation results provide important information for risk stratification. Therefore, variables from all three domains should be combined to provide the best prognostic analysis. Machine learning techniques significantly improves overall risk stratification performance.
