# Supplementary material for: Territory-wide cohort study of Brugada syndrome in Hong Kong: predictors of long-term outcomes using random survival forests and non-negative matrix factorisation
Source: Open Heart. 2021 Feb 5;8(1):e001505. doi: 10.1136/openhrt-2020-001505 (PMC7871343; doi:10.1136/openhrt-2020-001505)
Supplement: Supplementary data [file openhrt-2020-001505supp001.pdf]

Supplementary Tables

Supplementary Table 1. Data input into the random survival forest analysis. Variables included were: sex, age, initial type 1 Brugada pattern, Brugada pattern evolution, fever-induced type 1, family history of Brugada syndrome, family history of sudden cardiac death, syncope, prior VT/VF, positive electrophysiological study, positive genetic test, positive Holter findings, presence of other arrhythmias, heart rate, P-wave duration, PR interval, QRS interval, QTc interval, QT interval, P-wave axis, QRS axis, T-wave axis, RV5 amplitude, SV1 amplitude.

Feature	Value
Sample size	516
No. of patients with spontaneous VT/VF post-diagnosis	63
Data imputation	Yes
No. of trees	150
Forest terminal node size	15
Average no. of terminal nodes	12.55333
No. of variables at each split	5
Total no. of variables	24
Resampling used to grow trees	swor
Resample size used to grow trees	326
Family	surv
Splitting rule	logrank *random*
No. of random split points	10
Error rate	6.9%

Supplementary Table 2. Data input into the random survival forest analysis. Variables included were those in Supplementary Table 1, except for genetic test.

Feature	Value
Sample size	516
No. of patients with spontaneous VT/VF post-diagnosis	63
Data imputation	Yes
No. of trees	150
Forest terminal node size	15
Average no. of terminal nodes	10.96667
No. of variables at each split	5
Total no. of variables	23
Resampling used to grow trees	swor
Resample size used to grow trees	326
Family	surv
Splitting rule	logrank *random*
No. of random split points	10
Error rate	8.13%

Supplementary Table 3. Data input into the random survival forest analysis. Variables included were those in Supplementary Table 1, except for electrophysiological study.

Feature	Value
Sample size	516
No. of patients with spontaneous VT/VF post-diagnosis	63
Data imputation	Yes
No. of trees	150
Forest terminal node size	15
Average no. of terminal nodes	11.59333
No. of variables at each split	5
Total no. of variables	23
Resampling used to grow trees	swor
Resample size used to grow trees	326
Family	surv
Splitting rule	logrank *random*
No. of random split points	10
Error rate	7.32%

Supplementary Table 4. Data input into the random survival forest analysis. Variables included were those in Supplementary Table 1, except for genetic test and electrophysiological study.

Feature	Value
Sample size	516
No. of patients with spontaneous VT/VF post-diagnosis	63
Data imputation	Yes
No. of trees	150
Forest terminal node size	15
Average no. of terminal nodes	10.50667
No. of variables at each split	5
Total no. of variables	22
Resampling used to grow trees	swor
Resample size used to grow trees	326
Family	surv
Splitting rule	logrank *random*
No. of random split points	10
Error rate	7.07%

Detailed Methods

Clinical data was extracted from electronic health records. The following baseline clinical data were collected: 1) sex; 2) age of initial Brugada pattern (BrP) presentation; 3) follow-up period; 4) type of BrP and presence of fever at initial presentation; 5) family history of BrS and VF/ SCD; 5) manifestation of syncope and if present, the number of episodes; 6) manifestation of VT/VF and if present, the number of episodes; 6) performance of sodium channel blocker challenge test, electrophysiological study (EPS), 24-hours Holter study, BrS-related genetic screening, electroencephalogram (EEG) and respective results; 7) performance of treadmill test and echocardiogram; 8) concomitant presence of other arrhythmias (sick sinus syndrome, bradycardia, atrio-ventricular block, atrial tachy-arrhythmias, supraventricular tachy-arrhythmias) ; 9) implantation of ICD. Patients presented with two or more episodes of VT/VF were defined to be of high VT/VF burden. All types of syncope, including benign vagal syncope, were included. Bradycardia is defined as heart rate below 60 beats-per-minute. Atrio-ventricular block is defined as PR-interval greater than 200ms, dropped QRS complex or atrio-ventricular dissociation. Furthermore, the following details on the long-term outcomes were collected: 1) occurrence of death, and if so, the cause and age of death; 2) duration between initial BrP presentation and the first post-diagnosis VT/VF episode, if any. Data on pharmacological management of BrS through the use of amiodarone, disopyramide, isoprenaline, quinidine were obtained, including 1) indication; 2) treatment response; 3) presence of side effects; 4) dosing regimen [1]. Documented ECGs were reviewed to identify the presence of BrP evolution, and presentation of type 1 BrP anytime during follow-up. Evolution of ECG pattern is defined as the change in type of BrP presented, or the resolution of BrP, over the course of follow-up.

Positive results of different diagnostic tests were defined as follow. 1) Holter: detection of VT/VF or other cardiac arrhythmias (sick sinus syndrome, bradycardia, atrio-ventricular

block, atrial tachy-arrhythmias, supraventricular tachy-arrhythmias). 2) EPS: induction of spontaneous VT/VF lasting at least 30 seconds or producing hemodynamic collapse.

The study cohort was analyzed by initial symptom manifestation and initial Brugada pattern (BrP) type presentation. Initial symptom presentation is defined as the symptom presented when BrP was first identified, and the cohort is divided into three subgroups similarly: 1) asymptomatic; 2) syncope; 3) VT/VF. Asymptomatic is defined as the absence of syncope and ventricular tachyarrhythmia presentation. Patients with a history of syncope prior to the diagnosis of BrS is included in the syncope subgroup, since the possibility of missed BrP identification cannot be excluded. Based on initial BrP presentation, the cohort is categorized into three subgroups 1) type 1 BrP; 2) non-type 1 BrP; 3) non-type 1 BrP and evolved into type 1 BrP during follow-up.

ECG measurements

Automatically measured parameters from 12-lead ECG in standard position were extracted, including 1) heart rate; 2) P wave duration; 3) PR interval; 4) QRS duration; 5) QT and QTc interval; 6) P wave, QRS and T wave axis; 7) lead V1 S wave and lead V5 R wave amplitude. Besides from amplitude of lead V1 S wave and lead V5 R wave, the remaining ECG parameters were averaged across the 12 leads. If patients have more than one ECGs performed across different dates, the average and standard deviation (SD) of each index across the ECGs were calculated. SD was calculated to assess the temporal variability of the ECG indices.

Primary outcome, risk variables and statistical analysis

The primary outcome of this study was spontaneous VT/VF. Cox proportional hazard ratio regression with Efron's method for ties, and logistic regression were used to identify independent predictors for shorter time to the first post-diagnosis VT/VF event, and the occurrence of VT/VF during follow-up respectively. Significant predictors identified in univariate analysis were selected into the multivariate regression model. Separate models with the recruitment of predictors of P-value <0.005 and P-value<0.010 were formed. Multivariate analysis was repeated to include variables of investigations that were not performed across the entire cohort.

All statistical analysis was performed using Stata MP (Version 13.0). Categorical variables were expressed as total number (percentages) with intergroup differences obtained through Fisher's exact test. Continuous variables were expressed as mean ± standard deviation and compared by Kruskal-Wallis one-way ANOVA. These statistical tests were repeated with pairwise comparison of the groups performed when significant intergroup differences were found. P-value < 0.05 was considered statistically significant. Mean VT/VF incidence rate ratio per person-year for each subgroup was calculated by dividing the total number of VT/VF events by the multiple of the total follow-up period and the number of patients specific to the subgroup. Only sustained VT/VF episodes were included for the calculation of incidence rate ratio. Intergroup differences of VT/VF event rates were compared by incidence rate ratio two-sided exact significance test. Duration from the date of initial BrP presentation to the first post-diagnosis VT/VF event for patient subgroups of different initial presentation was compared qualitatively by Kaplan-Meier survival curve, and the intergroup differences were compared quantitatively by the log-rank test.

Random survival forest analysis and survival estimates

Random Survival Forest (RSF) analysis was performed to assess the relative importance of different predictors. RSF utilizes statistical methods to estimate the hazard function under the framework of a random forest [2] without making any assumptions about the individual hazard function [3], and ranks the significance of predictors for spontaneous VT/VF. The principles of the random survival forest model are shown in **Figure 2**. Features and samples are randomly selected for each single tree, and log-rank splitting is used to grow the trees. At the end of each branch, a cumulative hazard function is calculated for the selected individual trees. Finally, the ensembled estimated cumulative hazard function is computed by averaging the results of all the trees. The original dataset (n=516) was randomly divided into five equal-size subsets (n=103). Four subsets (n=413) were used as the training sets whereas the remaining subset was used as the validation dataset. The cross-validation process is repeated for five times. The results from the five validation processes were averaged to generate final predictions. RSF was trained using the RandomForestSRC R package (RStudio, Version 1.1.456). Survival estimates were calculated using the Brier score (0=perfect, 1=poor, and 0.25=guessing) based on the inverse probability of censoring weight (IPCW) method [4]. The cohort was stratified four groups of 0-25, 25-50, 50-75 and 75-100 percentile values of mortality.

Additional Results

Initial Symptoms

Patients were compared based on disease presentation at initial BrP presentation: 1) asymptomatic (n=314, initial BrP presentation age=50.9±15.7, follow-up duration=85.9±52.8 months); 2) syncope (n=161, initial BrP presentation age=49.3±16.5, follow-up

duration=87.2±51.9 months); 3) VT/VF (n=41, initial BrP presentation age=45.5±18.0, follow-up duration=89.7±63.9 months). Baseline characteristics with pairwise comparison in parameters with significant intergroup differences are presented on *Table 3A and 3B*. The mean VT/VF event rate per person-year differed significantly (P<0.0001), in descending order of VT/VF (1.70%), syncope (0.05%) and asymptomatic (0.01%). There is a significant intergroup difference in the time till VT/VF occur during follow-up (**Figure 1**; P-value: asymptomatic vs syncope<0.0001, asymptomatic vs VT/VF<0.0001, syncope vs VT/VF<0.0001), shortest time being the VT/VF group, followed by syncope and the asymptomatic group.

Similar to comparison based on overall disease manifestation, both average (P-value: asymptomatic vs syncope=0.278, asymptomatic vs VT/VF=0.015, syncope vs VT/VF=0.042) and baseline QTc interval (P-value: asymptomatic vs syncope=0.280, asymptomatic vs VT/VF=0.008, syncope vs VT/VF=0.033) are significantly longer in patients who presented with VT/VF initially. The standard deviation of QRS duration differed significantly by the descending order of VT/VF (11.5±12.4 ms), asymptomatic (8.13±9.17 ms) and syncope (6.35±5.12 ms) (P=0.004). The standard deviation in T-wave axis is significantly higher for the VT/VF group (P-value: asymptomatic vs syncope=0.346, asymptomatic vs VT/VF=0.042, syncope vs VT/VF=0.013). On the contrary, T-wave axis at baseline is the lowest for the VT/VF group (P-value: asymptomatic vs syncope=0.508, asymptomatic vs VT/VF=0.017, syncope vs VT/VF=0.044).

Initial ECG presentation

The baseline characteristics of patients with initial type 1 (n=319, initial BrP presentation age=49.6±16.0, follow-up duration=79.7±50.2 months) and non-type 1 BrP (n=197, initial BrP presentation age=50.5±16.5, follow-up duration=97.8±56.5 months)

presentation are displayed on *Table 5*. There is a significantly higher proportion of non-type 1 BrP patients (43.1%) who experienced an evolution in BrP presented than those presented with type 1 BrP initially (28.8%) (P=0.001). Amongst patients who evolved into type 1 BrP from an initial non-type 1 BrP, there was a higher portion of fever-induced patients than the overall initial non-type 1 BrP subgroup (P=0.019). Patients with initial type 1 BrP presentation are significantly more likely to have a family history of BrS (P=0.003) and SCD (P=0.044). Although the mean VT/VF incidence rate ratio per person-year differed significantly (type 1 BrP=0.003%, non-type 1 BrP=0.005%; P=0.000), there are no significant difference in the occurrence and number of syncope and VT/VF episodes between patients with initial type 1 and non-type 1 BrP throughout follow-up. Patients presented with non-type 1 BrP initially are more likely to have other underlying concomitant arrhythmias (P=0.024). In terms of the average value of ECG parameters, patients presented with type 1 BrP initially have higher heart rate (type 1 BrP=81.8±17.4 bpm, non-type 1 BrP=77.8±16.6 bpm; P=0.005) and shorter QT interval (type 1 BrP=369±36.8 ms, non-type 1 BrP=378±31.1 ms; P=0.009). However, there were no significant intergroup difference found for average QTc interval. Similarly, the type 1 BrP group also had higher baseline heart rate (type 1 BrP=83.0±20.6 bpm, non-type 1 BrP=77.2±18.4 bpm; P=0.008) and QT interval (type 1 BrP=365±45.1 ms, non-type 1 BrP=375±36.4 ms; P=0.014).

Mortality during follow-up

During follow-up, 37 patients passed away (asymptomatic=20, syncope=14, VT/VF=10, p=0.214). Of these, seven patients presented with both syncope and VT/VF. 23 patients presented with type 1 BrP initially whereas the remainder did not (P=1.00). 25 patients

were initially asymptomatic, whilst 9 presented with syncope, and 3 showed prior VT/VF at the time of diagnosis (P=0.639). Five of the 37 cases had BrS-related mortality (age of death=51.1±7.88, type 1 BrP=4). In terms of initial disease manifestation, two patients were asymptomatic, one patient presented with syncope, and two patients experienced VT/VF. It should be noted that one of the patients who passed away due to VF was suffering from pneumonia. Amongst the five patients, one had a family history of SCD, and another patient had both positive sodium channel blocker challenge test and inducible EPS. Two patients had EEG performed due to seizure presentation and found epileptic waveforms. One of these two patients also had underlying atrial fibrillation (AF). None of the patients had high VT/VF burden and did not have ICD implanted. One patient had a SCN5A genetic test performed and was tested negative.

Pharmacological therapy

A total of 50 patients were administered quinidine (average dose: 690±275 mg/day, average duration=48.1±55.3 days; type 1 BrP=45). One patient was prescribed the drug, but its use was withheld. The indications for quinidine use include: 1) VT/VF prevention before or alternative to ICD implantation (n=8); 2) VT/VF control (n=18); 3) recurrent appropriate ICD shock (n=7); 4) recurrent premature ventricular contraction (n=3); 4) prophylaxis for VT storm (n=2); 5) supraventricular tachycardia or AF control (n=1); 6) not recorded (n=10). Amongst patients who used quinidine for arrhythmia control, 28 patients were responsive to the drug whilst 9 patients were unresponsive. One patient terminated the course of drug despite positive response due to the adverse effect of skin rash and flushing. Two of the unresponsive patients were intolerant to the drug.

Amiodarone was administered to 40 patients (average dose=224±474 mg/day, average duration=22.6±43.7 days, type 1 BrP=29). The indications for amiodarone use were not recorded in 7 patients. The indication of drug use in the remaining patients include: 1) acute management of VT/VF (n=15); 2) long term VT/VF control (n=6); 3) appropriate ICD shock delivery (n=3); 4) AF (n=9). Amongst these 40 patients, 30 were responsive to amiodarone. Six patients experienced adverse effects, which include hypothyroidism (n=2), hyperthyroidism with palpitation (n=1), and non-specific discomfort. Two of the amiodarone-intolerant patients were switched to sotalol. For the remaining two non-responsive patients, one was switched to quinidine, and the other patient required cardioversion to manage the acute VT. Four of the six amiodarone-intolerant patients were administered amiodarone for long term VT/VF control.

A total of 11 patients were administered sotalol (average dose=96.6±47.5 mg/day, average duration=68.7±55.4 days; type 1 BrP=10). Besides from the four patients who switched from amiodarone to sotalol due to intolerance for long term VT/VF control, other indications for the drug administration include AF-induced inappropriate ICD shock (n=2) and underlying AF (n=1). Indications were not recorded for the remaining six patients. Five of the seven patients were responsive to sotalol, but the drug was terminated in two patients due to the adverse effect of dry cough and dizziness respectively. Amongst the remaining two patients, the drug was terminated in one patient due to bradycardia, and the other patient was switched to quinidine for VT/VF control.

Amongst the seven patients who were administered isoprenaline, two patients have known indications. One patient was prescribed for the tilt table test, and the other was responsive to its management of acute slow VT. One patient was administered and responsive to disopyramide when its dosage was increased from 100 mg to 400 mg daily for the management of appropriate ICD shock.

Genetic Information

Genetic mutations in the SCN5A gene identified in this study. DI, II, III or IV refers to domains 1 to 4 and S1, 2, 3, 4, 5 or 6 refers to segments 1 to 6 in the pore-forming protein subunit of the sodium channel encoded by SCN5A. Modified from [5] with permission.

SCN5A Mutation	Region in genome	Coding effect	Mutation type (by effect on DNA)	Location in SCN5A protein subunit	Novel mutation for BrS outside local territory (Y/N)
c.87G>A	Exon 2	A29A	Substitution	N-terminus	Y (likely normal variant)
c.429del	Exon 4	Asn144Thrfs*57	Deletion	DI-S1 (truncation)	Y
c.674G>A	Exon 6	R225Q	Substitution	DI-S4	Y
c.677C>T	Exon 6	A226V	Substitution	DI-S4	N
c.916C>T	Exon 8	L306F	Substitution	DI-S5-6	Y (likely normal variant)
c.1141-3C>A	Intron 9	-	Substitution (splice site)	-	N
c.1673A>G	Exon 12	H558R	Substitution	DI-DII	N
c.2024-11T>A	Exon 14	-	Substitution	DI-DII	Y
c.2042A>C	Exon 14	H681P	Substitution	DI-DII	Y

c.2893C>T	Exon 17	R965C	Substitution	DII-S6-DIII-S1	N
c.3578G>A	Exon 20	R1193Q	Substitution	DIII	N
c.4279G>T	Exon 24	A1427S	Substitution	DIII-S5/S6	Y
c.5350G>A	Exon 28	E1784K	Substitution	DIV-S4	N
c.5457T>C	Exon 28	D1819D	Substitution	C-terminus	N
c.5689C>T	Exon 28	R1897W	Substitution	C-terminus	Y
c.5851G>T	Exon 28	V1951L	Substitution	C-terminus	N

Further Discussions

Management approaches: observation, drugs and device therapy

Generally, there are three approaches to management of BrS patients, including observation with follow-up investigations, pharmacological therapy and device therapy. Pharmacological therapy aims to prevent ventricular arrhythmias by rebalancing the depolarization-repolarization imbalances across the myocardial wall. By contrast, device therapy aims to abort ventricular arrhythmias if they develop. Several drugs are used in BrS, including quinidine, disopyramide, amiodarone, beta adrenergic agonists, phosphodiesterase inhibitors, bepridil and amiodarone. Quinidine is an agent that blocks the transient outward potassium current, thereby normalizing the action potential dome in the epicardium and the ST segment. In our study, quinidine successfully prevented VT/VF in 68% patients (19/28) using an average dose of 690 mg. For secondary prevention, quinidine was prescribed for recurrent appropriate ICD shock (n=7), recurrent premature ventricular contraction (n=3) and VT storm (n=2). The success rates are comparable to those reported by previous studies. In a study using quinidine for shock reduction, 19 patients (66 %) remained free of appropriate ICD discharges

[6]. From our cohort, we found that three patients (6%) were intolerant to the drug because of side effects, which is lower than the 17% observed in this prior study. Disopyramide is a class IA antiarrhythmic drug with inhibitory action on the transient outward potassium current. It was only prescribed in one patient from our cohort for frequent ICD shocks. Amiodarone and sotalol were also used with good efficacy in our cohort. Finally, ICD is the mainstay treatment for both primary and secondary prevention of spontaneous VT/VF/SCD in BrS. In the present cohort, 26% of the patients had ICD implantation.

Random survival forest analysis

While statistical methods such as classification and regression trees may be intuitive for clinicians, they suffer from high variance and poor performance [7, 8]. These are addressed by RSF, which builds hundreds of tree branches and outputs the results by voting [2]. RSF reduces variance and bias by using all the collected variables, then automatically assess the nonlinear effects and complex interactions amongst the variables [3]. RSF is fully non-parametric, including the effects of the treatments and predictor variables, whereas traditional methods such as Cox model utilize a linear combination of attributes [9].

References

1. Brodie, O.T., Y. Michowitz, and B. Belhassen, *Pharmacological Therapy in Brugada Syndrome.* Arrhythm Electrophysiol Rev, 2018. **7**(2): p. 135-142.
2. Breiman, L., *Random Forests.* Machine Learning, 2001. **45**(1): p. 5-32.
3. Ishwaran, H., et al., *Random survival forests.* Ann. Appl. Stat., 2008. **2**(3): p. 841-860.
4. Gerds, T.A. and M. Schumacher, *Consistent estimation of the expected Brier score in general survival models with right-censored event times.* Biom J, 2006. **48**(6): p. 1029-40.
5. Tse, G., et al., *Identification of Novel SCN5A Single Nucleotide Variants in Brugada Syndrome: A Territory-Wide Study From Hong Kong.* Front Physiol, 2020. **11**: p. 574590.
6. Anguera, I., et al., *Shock Reduction With Long-Term Quinidine in Patients With Brugada Syndrome and Malignant Ventricular Arrhythmia Episodes.* J Am Coll Cardiol, 2016. **67**(13): p. 1653-4.
7. Hsich, E., et al., *Identifying important risk factors for survival in patient with systolic heart failure using random survival forests.* Circ Cardiovasc Qual Outcomes, 2011. **4**(1): p. 39-45.
8. Breiman, L., *Classification and regression trees*. 2017: Routledge.
9. Ishwaran, H., et al., *High-Dimensional Variable Selection for Survival Data.* Journal of the American Statistical Association, 2010. **105**(489): p. 205-217.
